# Analysing Breast Cancer Multidisciplinary Patient Management: A Prospective Observational Evaluation of Team Clinical Decision-Making

**DOI:** 10.1007/s00268-018-4815-3

**Published:** 2018-10-31

**Authors:** T. A. K. Gandamihardja, T. Soukup, S. McInerney, J. S. A. Green, N. Sevdalis

**Affiliations:** 1 Chelmsford Breast Unit, Broomfield Hospital, Chelmsford, Essex, UK; 2 Centre for Implementation Science, Health Service and Population Research Department, King’s College London, London, UK; 3 Charing Cross Hospital, Imperial College Healthcare NHS Trust, London, UK; 4 Department of Urology, Whipps Cross University Hospital, Barts Health NHS Trust, London, UK

## Abstract

**Background:**

Multidisciplinary team (MDT)-driven cancer care is a mandatory UK national policy, widely used globally. However, few studies have examined how MDT members make decisions as a team. We report a single-centre prospective study on team working within breast cancer MDT.

**Methods:**

This was a prospective observational study of 10 breast MDT meetings (MDM). Trained clinical observer scored quality of presented information and disciplinary contribution to case reviews in real time, using a validated tool, namely Metric for the Observation of Decision-Making. Data were analysed to evaluate quality of team working.

**Results:**

Ten MDMs were observed (*N* = 346 patients). An average of 42 patients were discussed per MDM (range: 29–51) with an average 3 min 20 s (range: 31 s–9 min) dedicated to each patient. Management decision was made in 99% of cases. In terms of contribution to case reviews, breast care nurses scored significantly (*p* < 0.05) lower (*M* = 1.79, SD = 0.12) compared to other team members (e.g. surgeons, *M* = 4.65; oncologists, *M* = 3.07; pathologists, *M* = 4.51; radiologists, *M* = 3.21). Information on patient psychosocial aspects (*M* = 1.69, SD = 0.68), comorbidities (*M* = 1.36, SD = 0.39) and views on treatment options (*M* = 1.47, SD = 0.34) was also significantly (*p* < 0.05) less well represented compared to radiology (*M* = 3.62, SD = 0.77), pathology (*M* = 4.42, SD = 0.49) and patient history (*M* = 3.91, SD = 0.48).

**Conclusion:**

MDT evaluation via direct observation in a meeting is feasible and reliable. We found consistent levels of quality of information coverage and contribution within the team, but certain aspects could be improved. Contribution to patient review resides predominantly with surgeons, while presented patient information is largely of biomedical nature. These findings can be fed to cancer MDTs to identify potential interventions for improvement.

## Introduction

The complexity of cancer diagnosis and treatment, together with the increasing need for individualised patient care, has led to the organisation of services into multidisciplinary teams (MDTs), otherwise termed tumour boards or cancer conferences. The UK Department of Health defines MDT as “*a group of people of different healthcare disciplines which meets together at a given time (whether physically in one place or by video or teleconferencing) to discuss a given patient, and who are each able to contribute independently to the diagnostic and treatment decisions about the patient*” [1]. In the UK, the practice of undertaking cancer MDT meetings (or, MDMs) started following the Calman–Hine report in 1995, which found a variation in the delivery of cancer care [2] and was further driven by the launch of the National Health Service (NHS) Breast Cancer Screening programme in 1998. MDTs are now a mandatory component of cancer care and are regulated through the annual peer review process, which ensures adherence to national tumour-specific guidelines with the aim to standardise and improve outcomes of cancer patients [3]. Outside the UK, the MDTs are becoming increasingly prevalent globally.

Within breast cancer care, the importance of MDTs and MDMs has been further emphasised by guidelines set out by the Association of Breast Surgery (ABS) [4] and further guidelines jointly produced with the British Association of Plastic and Reconstructive and Aesthetic Surgeons (BAPRAS) [5]. As a result, in breast cancer services, at a minimum, the MDT consists of the core members, including, clinical and medical oncologists, breast surgeons, radiologists, pathologists and breast care nurse specialists (BCNs) [1, 3]. These key professionals meet, usually once a week, to review patients’ clinical details, their imaging and pathology results, and formulate an individualised management plan [6].

The benefit of an MDT is provision of coordinated, consistent, expert-driven and cost-effective care to the patient [7]. The MDMs are assumed to improve communication between different disciplines, facilitate decision-making and ensure adherence to clinical guidelines [6–8]. It also allows collection of data for audit and research purposes, identification of suitable patients for clinical trials and serves as an educational forum. However, the evidence base to support the beneficial effect of MDT care on breast cancer patient survival and outcomes has produced variable results [9–12].

The assumption that an MDT will make the best decision on cancer care depends upon a variety of factors, all of which apply to expert teams in general. These include availability of appropriate information to facilitate decision-making process, good team working, attendance of the core members and effective leadership [13–15]. Although MDT-driven care is the norm in cancer services, to date there are very few studies systematically investigating the efficacy of team working in cancer MDT setting generally [16, 17], and breast cancer in particular. For other specialities, evidence shows unequal contribution to patient review leaning towards surgical and oncological disciplines with patient information being of predominantly biomedical nature and patient psychosocial aspects, their views on treatment options and their comorbidities underrepresented [6, 7, 13, 14, 16–23].

The aim of this study is therefore twofold. First, to explore feasibility of assessing the quality of decision-making using a novel observational tool [18], and second, to test the previous finding in a new context of a breast cancer MDT. We therefore hypothesise that patient information relating to medical history, radiology and pathology will be better presented in comparison with patients’ views on treatment options, their comorbidities and psychosocial circumstances (hypothesis 1, H1). We also predict that the disciplinary contribution to patient reviews will be unequal with the highest input from surgeons and the lowest from breast care nurses (hypothesis 2, H2).

## Methods

### Participants and setting

This is a prospective observational study, undertaken in the breast cancer MDT of an inner-city teaching hospital in London, UK. The hospital treats over 500 breast cancer patients each year. It is also a breast screening unit, receiving patients referred by the NHS breast screening programme. All patients who have been seen within the breast unit with a confirmed or suspected breast cancer are reviewed at the MDM, which occurs once a week.

The study was conducted in real time over 10 consecutive weekly MDMs. All patients who were reviewed during these meetings were included in the study, with no exclusions. The core members of the MDT included breast surgeons, clinical and medical oncologists, radiologists, histopathologists and breast care nurses (BCNs).

This study was granted Ethical Approval by the local ethics committee prior to data collection (JRCO REF. 157441).

### Materials

**Fig. 1:**
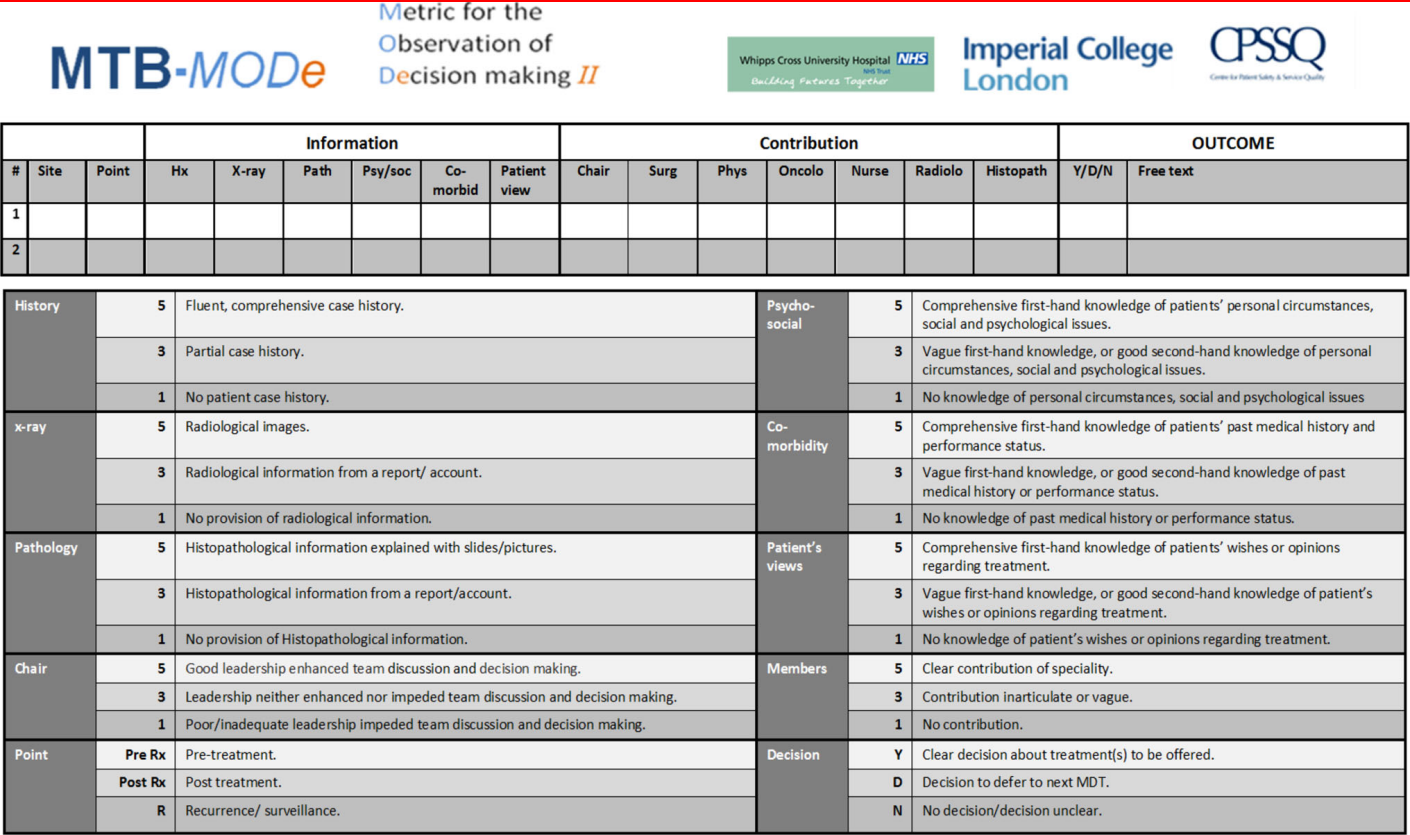
Metric for the observation of decision-making in cancer multidisciplinary team meeting (MDT-MODe)

We used a previously validated observational assessment tool specifically designed for cancer MDMs, namely the Metric for the Observation of Decision-Making [18] (MDT-MODe; see Fig. 1). This instrument was developed and validated by our research team and has been used to investigate and improve efficacy of team working within other cancer MDMs [16, 18, 19, 22, 23].

The MDT-MODe [18] captures the following aspects in a meeting:

1. *Quality of presented patient information* which includes six individual variables scored on a behaviourally anchored five-point scale, namely patients’ case history, radiological images, histopathology, psychosocial issues, comorbidities and their views on treatment options. The sum of the scores for all six variables represents overall quality of presented information for a patient with the higher scores indicating better quality.
2. *Quality of disciplinary contribution to patient reviews* which includes six individual variables scored on a behaviourally anchored five-point scale, representing the surgeons, oncologists, radiologists, histopathologists, BCNs and the chairperson. However, there was no formally appointed meeting chair in the participating team, and so this variable was not scored and analysed. The sum of the scores for all six variables represents overall quality of disciplinary contribution for a patient with the higher scores indicating better quality.
3. *Team ability to reach treatment recommendation for a patient* which is a categorical variable allowing one to note whether the decision was made or not, but also whether it was deferred.

### Assessor training

Prior to the study, a dedicated clinical observer (SM; senior breast care nurse specialist) was trained in the use of the MDT-MODe. This involved watching videos of MDMs and mock scoring to ensure proficiency in the use of the tool.

During the study, three MDT meetings were double-rated blindly between the clinical and psychologist evaluators to ensure inter-observer reliability and accurate use of the tool. The observers did not participate clinically in the meeting. The main clinical observer was blind to the study hypothesis.

### Statistical methods

All analyses were conducted using SPSS^®^ v 20.0. For the inter-observer reliability, we used the standard recommended interclass correlation coefficients (ICCs); these range between 0 and 1, with higher values indicating better agreement between evaluators. Coefficients of 0.70 or higher across observations are considered adequate for such observational assessments [24]. For hypotheses testing, we used a related sample Wilcoxon signed-rank test.

## Results

### Meeting characteristics

A total of 346 patient reviews were assessed live during 10 consecutive weekly MDMs. The characteristics of individual meetings are illustrated in Table 1. On average, 42 patients were discussed per meeting, lasting just over 3 h with an average of 3 min and 20 s dedicated to each patient.

**Table 1:** Meeting characteristics of the breast cancer multidisciplinary team

Meeting characteristics	Mean	Min.	Max.	Sum
Number of meetings observed	–	–	–	10
Number of patient discussions per meeting	42	29	51	346^a^
Number of symptomatic patient reviews per meeting	16	7	24	122
Number of postoperative patient reviews per meeting	26	20	32	224
Number of core members present per meeting	11	10	13	18^b^
Meeting duration (h:min:s)	03:05:00	02:45:00	03:30:00	–
Time per patient review (h:min:s)	00:03:20	00:00:31	00:09:00	–

^a^Sum of patients discussed over 10 observed meetings

^b^Total number of team members

### Reliability of evaluations

34% of the study sample (i.e. three meetings or 116 patient reviews) was double-rated blindly revealing overall very good reliability (>0.70) of observations between observers (ICCs ranged between 0.73 and 0.93 across the variables). For the quality of information presented, inter-assessor reliability was high with the ICCs ranging between 0.81 and 0.92, while for the quality of disciplinary contribution inter-assessor reliability was good to high with the ICCs from 0.73 to 0.90.

### Quality of presented patient information

Descriptive statistics are shown in Table 2. The Wilcoxon test shows that patient history (*Z* = − 14.62, *Z* = 15.33, *Z* = − 15.07, respectively), radiological (*Z* = − 12.15, *Z* = − 13.19, *Z* = − 123.36, respectively) and pathological information (*Z* = − 15.27, *Z* = − 15.86, *Z* = − 15.38, respectively) were significantly higher scored than psychosocial information, patient comorbidities and patient views (all comparisons significant, *ps *< 0.001). In contrast, while there was no significant difference between quality of presentation of patient history and radiology (*Z* = − 1.92, *p *> 0.05), they were both significantly lower in comparison with pathology (*Z* = 5.29, *Z* = 5.00, respectively, both comparisons significant at *p* < 0.001). There was no statistical difference between psychosocial information and comorbidities (*Z* = − 2.00) and patient views (*Z* = − 1.70), as well as between comorbidities and patient views (*Z* = 0.64; all *ps *> 0.05). These analyses support the H1.

**Table 2:** Descriptive statistics for the metric of decision-making (MDT-MODe) variables

MDT-MODe variables	Mean	SD	Median	Min–Max
Quality of information presentation				
Patient history	3.91	0.48	4	1–5
Radiology	3.62	0.77	5	1–5
Pathology	4.42	0.49	5	1–5
Psychosocial	1.69	0.68	1	1–5
Comorbidity	1.36	0.39	1	1–5
Patient views	1.47	0.34	1	1–5
Quality of contribution to patient reviews				
Chair^a^	1.00	–	–	–
Surgeon	4.65	0.56	5	1–5
Oncologist	3.07	0.58	3	1–5
Nurse	1.79	0.12	1	1–5
Radiologist	3.21	0.47	5	1–5
Pathologist	4.51	0.40	5	1–5

Scores for other individual items range from 1 to 5; higher scores indicate better quality of information and contribution to case review

^a^Score for chair is constant, receiving a score of 1 throughout the study due to the team not having a formally appointed chair in the meeting

### Quality of disciplinary contribution to patient reviews

Descriptive statistics are shown in Table 2. The Wilcoxon test shows that surgeons scored significantly higher in comparison with oncologists (*Z* = − 11.29, *p* < 0.001), BCNs (*Z* = − 15.35, *p* < 0.001), radiologists (*Z* = − 9.94, *p* < 0.001) and pathologists (*Z* = − 2.05, *p* < 0.05). Oncologists scored significantly higher than BCNs (*Z* = − 8.88, *p* < 0.001), but significantly lower than pathologists (*Z* = 9.81, *p* < 0.001), with no difference in comparison with radiologists (*Z* = 1.51, *p *> 0.05). BCNs scored significantly lower than radiologists (*Z* = 9.54, *p* < 0.001) and pathologists (*Z* = 14.31, *p* < 0.001). Radiologists scored significantly less than pathologists (*Z* = 7.61, *p* < 0.001). These analyses support the H2. However, there was no formal meeting chair in the team, and so the “chairing and leadership” element of the MDT-MODe did not receive any scores.

### Team ability to reach treatment recommendation for a patient

A treatment recommendation was made in 92.2% of patients (*N* = 319), while 6.4% (*N* = 22) received a deferred decision, and 21% (*N* = 72) were not known to the MDT members prior to the meeting (see Table 3). The 22 decisions that were deferred still had an outcome but not a definitive management plan. These decisions would include scenarios such as patients needed to be re-discussed due to incomplete pathology results or pending imaging. For 1.5% of patients (*N* = 5), no decision was reached at the MDM, which may reflect the inability of the observers to confidently note an outcome due to lack of clarity, such was the case for eight patients in total.

**Table 3:** Team ability to reach a decision outcome for patients reviewed in breast cancer meetings

Decision made	Observer is aware of treatment decision			Patient has been seen by a team member prior to the meeting		
	Total	Yes	No	Yes	No	Inconclusive
Yes	319	317	2	244	68	7
No	5	2	3	3	2	0
Deferred	22	18	4	19	2	1
Total	346	337	9	266	72	8

## Discussion

The current study set out to (1) determine feasibility of assessing team working in a breast cancer MDT, and (2) investigate two hypotheses stemming from earlier work in MDTs [6, 7, 13, 14, 16–23], namely suboptimal information sharing (H1) and unequal disciplinary input (H2). We demonstrate that it is feasible to quantitatively and prospectively measure how well a breast cancer MDT performs using a validated observational tool. We also demonstrate support for the two hypotheses. Specifically, the significantly higher scores on the presentation of the biomedical aspects of the diseases (radiology, pathology and medical history) are compared to patients’ psychosocial aspects, comorbidities and views on treatment options (H1), as well as significantly higher scores for surgeons (who gave the highest quality of input), pathologists, radiologists and oncologists as opposed to BCNs (H2). Despite this pattern of variability, the team had made treatment recommendations for 94% of patients.

The MDT model of care has become an integral part of delivery of cancer care in many countries globally, and mandatory in the UK [1]. However, there exists variability in how MDMs are organised and run [2]. Research has shown that they can be reliably evaluated through observational studies [16–19, 22, 23], and, to our knowledge, this is the first published study that evaluates performance of MDMs within the specialty of breast cancer, demonstrating feasibility and reliability of such approach.

Echoed by other MDT studies of different tumour types, where lack of consideration of patient-related factors and unequal disciplinary input to patient reviews has been shown [14, 19], current study demonstrates the same pattern. While our findings may reflect that most breast cancer patients are usually well and healthy at the time of their diagnosis, these remain important areas requiring attention. This is especially true when patient-related factors at MDMs are not considered, which may result in non-implementation of MDT recommendations [15]. It is thus arguable that an improved quality of information presented and contributions made by the MDT members lends itself to a more comprehensive and holistic discussion of each patient, which in turn may translate into a more efficient MDT with increased satisfaction of the involved healthcare professionals, an important measure of healthcare effectiveness [20]. Further quality-improving work in breast cancer MDTs is thus essential.

### Limitations

However, our study findings need to be interpreted within certain limitations. First is the Hawthorne effect, i.e. people changing behaviour due to being observed, which is a natural limitation to all observational studies. To help minimise this effect, we had a breast care nurse specialist as the main observer because their presence in the meetings is expected. The second limitation is observer bias, which could have impacted the study findings. While the main observer was blind to the study hypothesis, the second observer was not. We have, therefore, ensured that a cross section of patient reviews is double-rated with adequate inter-rater reliability, thus minimising the effect.

Third, this study was conducted in a single large teaching hospital, which may not represent other sites, although the core members of the MDT would be replicated in most if not all other MDT settings in the UK, as well as in other countries. The large number of patients reviewed at each meeting in the study also offers some representation of the usual MDT load, while the patients comprising both, symptomatic and screening groups, are also representative of other hospitals. However, the distribution of patients along the care pathway could have affected which information was presented as well as the information quality. This is a natural limitation to studies such as ours, which rely on naturalistic observations of teams and processes in real time with no manipulation of variables. There is no ability to control or influence extraneous factors potentially impacting the observed phenomenon.

Lastly, the tool that we used is an observational assessment instrument that relies on human judgment for the measurement data. As such, we have encountered certain learning points that could be of benefit to other scientists doing similar work with cancer teams. For example, (1) silence in the meetings can be difficult to interpret, (2) unsystematic presentation of information and contribution into the discussion can lead to difficulties in differentiating individual variables when scoring, and (3) scoring a meeting for a prolonged period of time can be cognitively taxing for the observer.

### Implications

Our results have implications for the working and structuring of the breast cancer MDTs, and as such warrant further research to help drive quality improvement and streamline team processes. For example, engagement of BCNs in patient reviews in MDMs is essential, since, as patients’ advocates, they are best placed to present patients’ views, ensuring that a proposed treatment plan has incorporated them [22, 26, 27]. This is important because patient choice has been shown to be the commonest reason for an MDT decision to be altered in breast cancer patients [21–23].

Various quality-improving strategies could be used to help facilitate disciplinary engagement within an MDT and ensure that all necessary patient information is presented in the meetings. For example, *changing the room layout* from rows of chairs (such is the case in the current study, and common in other settings) to a U-shape configuration helps diminish perceived hierarchies and encourage participation [25]. *A checklist* may further facilitate in this process [26]—structuring the team’s routine to consider some typically omitted aspects of patient care such as psychosocial element or patient’s wishes (as in the current study). *Appointing a chairperson* (possibly a rotational chair to promote team working) can also help by ensuring that all voices are heard and all necessary information shared [27]. Future studies could profitably test efficacy and feasibility of these quality-improving interventions and help streamline team processes.

Lastly, while in the current study a treatment plan was made for the large majority of patients (94%), we have not assessed quality of these decisions. Nonetheless, this is an important avenue of research since many elements can affect quality of decisions, such as, for example, unfamiliarity of the MDT with the patient, lack of leadership and time pressures [14, 15]. Further studies are therefore needed, particularly within the specialty of breast cancer.

### Conclusion

Current study demonstrates feasibility of assessing the performance of a breast cancer MDT. The quality of disciplinary contributions to patient reviews, as well as the presented patient information was measured in a quantitative manner using a validated observational assessment tool. The findings showed variability across the types of clinical information available and disciplinary inputs to the team’s decision-making. Specifically, contribution to discussion predominantly resided with surgeons, and presented patient information was largely of a biomedical content. These findings corroborate previous research and extend it into breast cancer care. Objective, structured assessment such as the one reported here can enable identification of areas for improvement within the MDT and drive quality improvement interventions aimed at streamlining team processes. Further studies are needed to determine efficacy and feasibility of such interventions.
